# Evaluating the impact of electronic whiteboard icons: an observational study of the work with blood tests in an emergency department

**DOI:** 10.1186/1757-7241-21-S2-A26

**Published:** 2013-09-09

**Authors:** Arnvør á Torkilsheyggi, Morten Hertzum, Gustav From

**Affiliations:** 1 Computer Science and Informatics, Roskilde University, Roskilde, Denmark; 2 Emergency Department, Slagelse Hospital, Slagelse, Denmark

## Background

Results of blood test are essential and often guiding for the diagnostic work. In emergency departments (EDs) a competent and rapid treatment therefore requires an efficient process for ordering blood tests, informing clinicians that samples have been taken, for communicating test results, and for physicians acknowledging having assessed results.

The ED in Slagelse Sygehus has implemented icons on their electronic whiteboard that visualize the progress of the blood-test process.

The aim of our study was to evaluate the impact of the icons on the workflow.

## Methods

The study was designed as a qualitative study using the methods of observations and informal interviews.

The observations amounted to 19 hours in total and consisted of shadowing 6 physicians and 2 nurses for a couple of hours at a time. Informal individual interviews were held with all observed professionals and with another two nurses, two laboratory technicians, a coordination nurse, a triage nurse, and a secretary.

## Results

The nurses frequently attended to the icons on the whiteboard. The arrival of new test results was seen as an opportunity to make the physicians aware of patients that could be discharged or transferred to other departments. The icons thereby supported the nurses in maintaining flow of patients.

The physicians did not attend to the icons for maintaining flow, but used the icons at two daily timeouts, when they collectively assessed patients. Overall they considered test results as input to the clinical evaluation of individual patients.

## Conclusion

Our study indicates that the blood-test process can contribute to the steering of which patient the physicians should see next. Presently, this steering is mediated by nurses, who keep an eye out for the icons reflecting the blood-test process.

The physicians did not attend the icons themselves, hence the blood-test process did not steer physicians directly. If the blood-test process should steer physicians’ actions directly, bypassing the nurses, the information of blood tests should be mediated by other means, for example by smartphones carried by the physicians. This calls for further studies.

